# Gastric Cancer Metastasis to the Prostate Detected on 18F-Fluorodeoxyglucose Positron Emission Tomography/Computed Tomography (18F-FDG PET/CT): A Report of a Rare Case

**DOI:** 10.7759/cureus.88048

**Published:** 2025-07-16

**Authors:** Ryo Sato, Asuka Uchiyama, Daisuke Suzuki, Yukihiro Yoshimi, Tetsuharu Nishio, Yu Matsunaga, Rikiya Matsumoto

**Affiliations:** 1 Department of Urology, Chutoen General Medical Center, Kakegawa, JPN; 2 Department of Urology, Hamamatsu University School of Medicine, Hamamatsu, JPN; 3 Department of Pathology, Chutoen General Medical Center, Kakegawa, JPN

**Keywords:** 18f-fdg-pet/ct, metastasis, prostate biopsy, prostate metastasis, prostate mri

## Abstract

We report an exceptionally rare case of prostate metastasis in a 70-year-old man with a history of gastric cancer. Following distal gastrectomy (pT4aN0M0) and one year of adjuvant chemotherapy with S-1, he remained recurrence-free until a gradual rise in serum carbohydrate antigen 19-9 was observed, reaching 1878.97 U/mL. Contrast-enhanced computed tomography and esophagogastroduodenoscopy failed to identify a site of recurrence. However, ^18^F-fluorodeoxyglucose positron emission tomography/computed tomography (^18^F-FDG PET/CT) detected abnormal uptake at the apex of the prostate, with a maximum standardized uptake value of 11. Magnetic resonance imaging was non-contributory, and serum prostate-specific antigen (PSA) was mildly elevated at 6.04 ng/mL. A transrectal ultrasound-guided prostate biopsy revealed poorly differentiated adenocarcinoma. Immunohistochemistry was negative for PSA and NKX3.1, but positive for cytokeratin 7, and histological comparison with the prior gastric specimen confirmed metastatic gastric cancer to the prostate. This case underscores the diagnostic challenge posed by rare metastatic patterns and highlights the complementary value of ^18^F-FDG PET/CT in detecting occult lesions when conventional imaging fails. In patients with a history of gastric cancer and rising tumor markers, early consideration of PET/CT followed by histological confirmation may facilitate timely and accurate diagnosis.

## Introduction

Prostate metastasis from gastric cancer is exceedingly rare, with fewer than 15 cases reported in the literature to date [1-3]. While secondary tumors of the prostate are uncommon, accounting for approximately 0.2% of all prostate malignancies according to surgical, biopsy, and autopsy series, the stomach is an especially rare primary source [4]. Gastric cancer commonly metastasizes to regional lymph nodes, the liver, or the peritoneum, while involvement of the prostate is highly unusual [5].

In most reported cases, patients typically presented with urinary symptoms that prompted urological evaluation [6]. However, metastatic lesions to the prostate may mimic primary prostate cancer both clinically and radiologically. This is especially true when prostate-specific antigen (PSA) levels are only mildly elevated and magnetic resonance imaging (MRI) findings are non-diagnostic. These overlapping features pose significant diagnostic challenges, as misidentifying the lesion may result in inappropriate local therapy or delayed systemic treatment.

Here, we present a rare case of prostatic metastasis from gastric cancer that was initially suspected based on elevated serum carbohydrate antigen 19-9 (CA19-9) and ^18^F-fluorodeoxyglucose positron emission tomography/computed tomography (^18^F-FDG PET/CT) findings and ultimately diagnosed via prostate biopsy and immunohistochemical comparison with the primary tumor.

This case highlights not only the diagnostic complexity of such presentations but also the utility of PET/CT in detecting rare metastatic patterns in patients with inconclusive conventional imaging such as MRI. It reinforces the need for a multidisciplinary approach when evaluating prostate lesions in patients with a history of gastrointestinal malignancy.

## Case presentation

A 70-year-old man was previously diagnosed with gastric cancer and underwent open distal gastrectomy. The pathological diagnosis was pT4aN0M0. He completed one year of adjuvant chemotherapy with S-1, finishing approximately one year before the current evaluation. During routine follow-up, he remained free of clinical or radiological evidence of recurrence.

**Figure 1:**
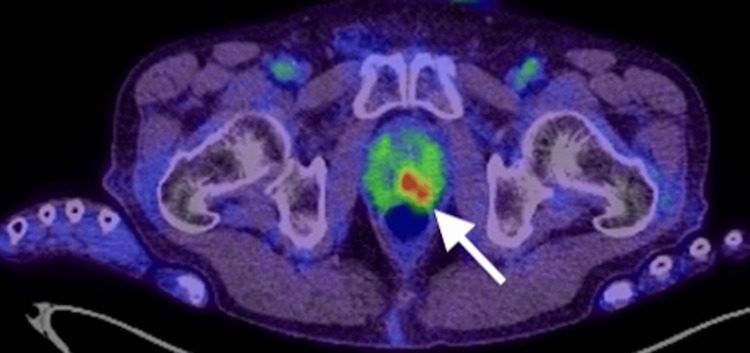
18F-FDG-PET/CT scan The abnormal uptake of ^18^F-FDG is observed at the apex of the prostate (white arrow), with a SUVmax of 11. ^18^F-FDG-PET/CT: ^18^F-fluorodeoxyglucose positron emission tomography/computed tomography; SUVmax: maximum standardized uptake value

Approximately six months prior to presentation, his serum CA19-9 level began to rise gradually. At the time of presentation to our department, the CA19-9 had increased markedly to 1878.97 U/mL (normal range: <37 U/mL). Despite this elevation, neither contrast-enhanced CT nor esophagogastroduodenoscopy (EGD) revealed any definitive signs of recurrence or metastasis. Subsequently, ^18^F-FDG PET/CT demonstrated a focal area of abnormal uptake at the apex of the prostate, with a maximum standardized uptake value (SUVmax) of 11 (Figure 1). Notably, he did not report any lower urinary tract symptoms, such as dysuria, frequency, urgency, or hematuria.

**Figure 2:**
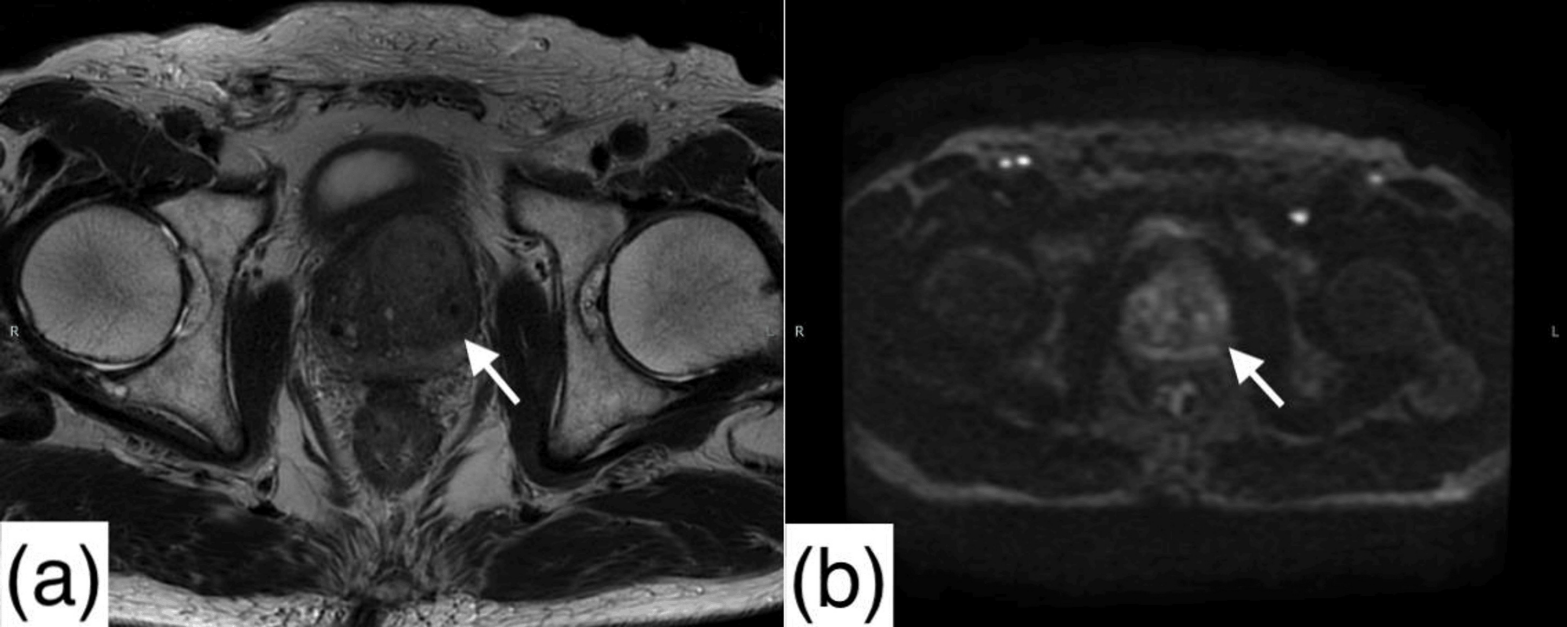
Magnetic resonance imaging of the pelvis (a) T2-weighted sequence reveals no apparent abnormal signal intensity within the prostate (white arrow). (b) Diffusion-weighted sequence reveals no apparent abnormal signal intensity within the prostate (white arrow).

MRI of the prostate did not reveal any distinct abnormal signal (Figure 2), but the serum PSA level was mildly elevated at 6.04 ng/mL (normal range: ≤4 ng/mL). Considering the possibility of primary prostate cancer or metastatic gastric cancer to the prostate, a transrectal ultrasound-guided prostate biopsy was performed.

**Figure 3:**
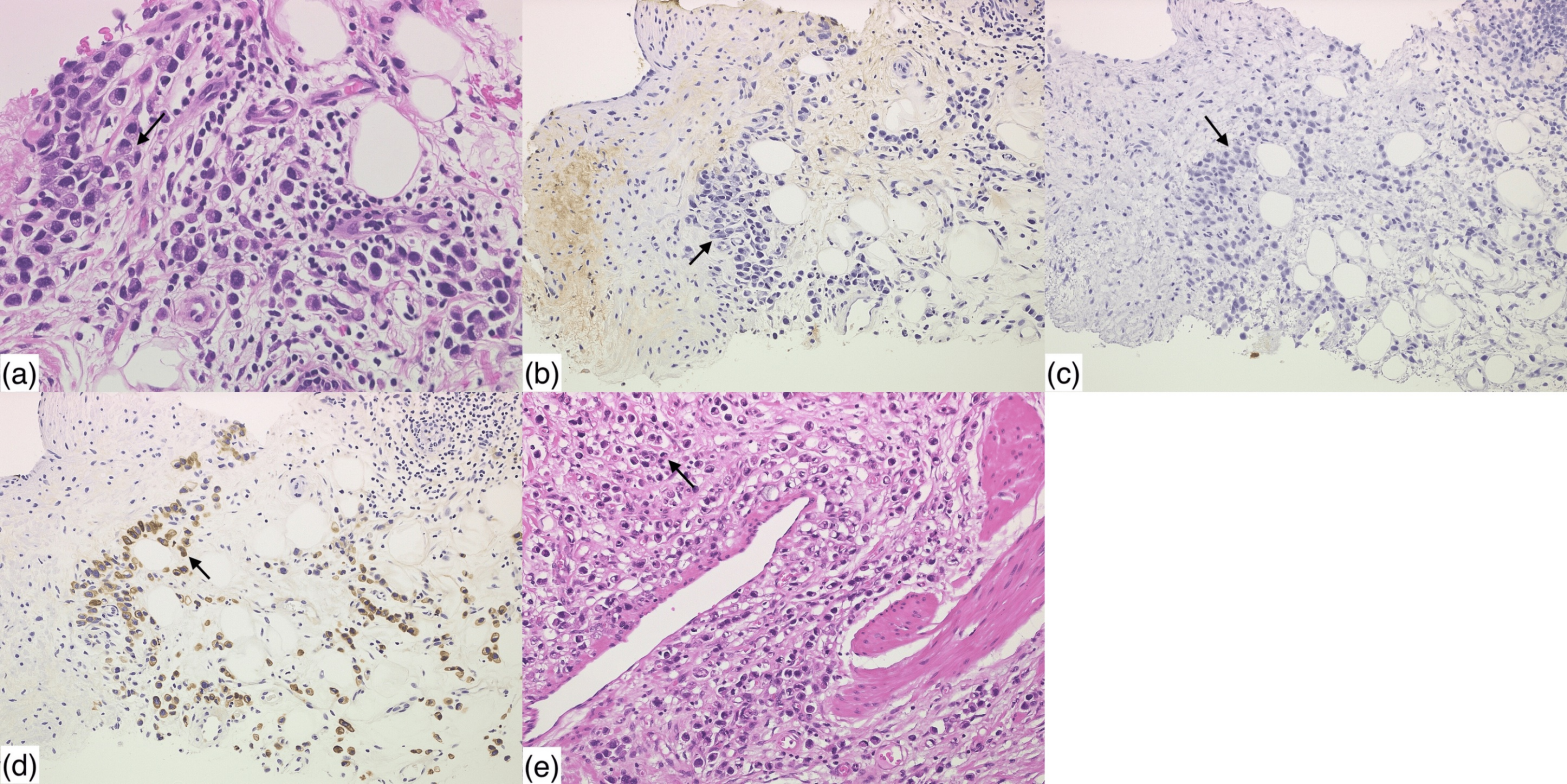
Histopathology (a) The prostate biopsy specimen shows poorly differentiated adenocarcinoma (black arrow), consistent with metastasis from the gastric cancer. (b) The prostate biopsy specimen shows negative staining for prostate-specific antigen on immunohistochemical analysis (black arrow). (c) The prostate biopsy specimen shows negative staining for NKX3.1 on immunohistochemical analysis (black arrow). (d) The prostate biopsy specimen shows positive staining for cytokeratin 7 on immunohistochemical analysis (black arrow). (e) The primary gastric cancer exhibits poorly differentiated adenocarcinoma (black arrow).

Histopathological examination revealed poorly differentiated adenocarcinoma (Figure 3a). Immunohistochemical staining showed the tumor cells were negative for PSA (Figure 3b) and NKX3.1 (Figure 3c) but positive for cytokeratin 7 (CK7) (Figure 3d). A comparative analysis with the patient's previous gastric cancer specimen (Figure 3e) revealed morphological similarities, leading to a final diagnosis of prostate metastasis from gastric adenocarcinoma. The patient is currently receiving multidisciplinary treatment at another institution.

## Discussion

Although gastric cancer metastasis to the prostate is rare, diagnosis can be challenging when clinical and imaging findings are nonspecific. In this case, the absence of urinary symptoms and the mild elevation of PSA initially suggested the possibility of early-stage primary prostate cancer. However, prostate MRI revealed no distinct lesion, further complicating the diagnosis.

Both PSA and MRI are widely used in the evaluation of suspected prostate cancer, but each has important limitations. PSA shows high sensitivity (93%) but low specificity (20%) and may be elevated in benign or non-prostatic malignant conditions [7]. MRI also has limited accuracy, especially in patients with PSA levels of 4-10 ng/mL, with pooled sensitivity and specificity of 84% and 76%, respectively, for clinically significant cancer [8]. In our case, these limitations contributed to diagnostic uncertainty, necessitating histological confirmation.

Notably, ^18^F-FDG PET/CT played a critical role in identifying the prostate lesion in our case. While ^18^F-FDG PET/CT is not routinely used for primary prostate cancer due to low uptake in typical cases, it can be useful for detecting metastases from tumors with higher glucose metabolism, such as gastric cancer. According to a recent review, the sensitivity and specificity of ^18^F-FDG PET/CT for detecting primary prostate cancer vary widely, with reported sensitivity ranging from 4% to 80% and specificity from 62% to 100%, depending on tumor grade, lesion location, and patient selection [9]. In our case, the modality successfully identified a metabolically active lesion within the prostate, guiding further evaluation.

Histopathological examination with immunohistochemistry was essential for establishing the diagnosis. The tumor was negative for prostate-specific markers (PSA and NKX3.1) and positive for CK7, a finding not specific to gastric cancer but also seen in other malignancies such as colorectal adenocarcinoma. However, no colorectal lesions were identified on imaging or endoscopy. Moreover, histological comparison with the patient's prior gastric cancer specimen revealed morphological similarity. Taken together, these findings made primary prostate cancer and colorectal metastasis unlikely, supporting a final diagnosis of prostatic metastasis from gastric cancer.

This case differs from previously reported cases of gastric cancer metastasizing to the prostate in several notable ways: the patient lacked urinary symptoms, had only a mildly elevated PSA, and showed no abnormalities on MRI. These features highlight the diagnostic value of PET/CT and suggest that this modality may be underutilized in the surveillance of patients with a history of gastric cancer and isolated tumor marker elevation.

In terms of clinical management, early histological evaluation is essential when imaging is inconclusive and the clinical presentation is atypical. Accurate identification of metastatic disease enables appropriate oncologic decision-making and avoids unnecessary local treatment for presumed primary prostate cancer.

## Conclusions

Prostate metastasis from gastric cancer is extremely rare and often presents with nonspecific findings. In patients with a history of gastric cancer and unexplained tumor marker elevation, clinicians should consider the possibility of atypical metastatic spread, even in the absence of urinary symptoms or definitive imaging abnormalities. Early utilization of ^18^F-FDG PET/CT may help identify metabolically active lesions that are not evident on conventional imaging.

Definitive diagnosis requires histopathological confirmation with immunohistochemical analysis, especially to distinguish metastatic disease from primary prostate cancer or other malignancies. In such atypical presentations, prompt biopsy should be pursued when imaging is inconclusive. This case highlights the importance of integrating tumor marker trends, advanced imaging, and tissue diagnosis in managing patients with suspected recurrence and may inform diagnostic strategies for similar clinical scenarios.

## References

[1] Zhang P, Zheng Y, Ran H, Leng Z, Wang Z (2010). Case report: gastric adenocarcinoma metastatic to the prostate gland. J Radiol Case Rep.

[2] Roshni S, Anoop T, Preethi T, Shubanshu G, Lijeesh A (2014). Gastric adenocarcinoma with prostatic metastasis. J Gastric Cancer.

[3] Shimizu N, Kawamoto B, Muraoka K (2025). Metastatic gastric adenocarcinoma in prostate: a case report. Urol Case Rep.

[4] Erdem H (2019). Secondary tumors of the prostate. J Urol Surg.

[5] Verstegen MH, Harker M, van de Water C (2020). Metastatic pattern in esophageal and gastric cancer: influenced by site and histology. World J Gastroenterol.

[6] Aşman EE, Ertunç O, Akdeniz R, Eryılmaz K (2024). Prostate metastasis from gastric malignancy: a rare case report and literature review. Bull Urooncol.

[7] Merriel SW, Pocock L, Gilbert E, Creavin S, Walter FM, Spencer A, Hamilton W (2022). Systematic review and meta-analysis of the diagnostic accuracy of prostate-specific antigen (PSA) for the detection of prostate cancer in symptomatic patients. BMC Med.

[8] Guo E, Xu L, Zhang D (2024). Diagnostic performance of MRI in detecting prostate cancer in patients with prostate-specific antigen levels of 4-10 ng/mL: a systematic review and meta-analysis. Insights Imaging.

[9] Shen K, Liu B, Zhou X, Ji Y, Chen L, Wang Q, Xue W (2021). The evolving role of 18F-FDG PET/CT in diagnosis and prognosis prediction in progressive prostate cancer. Front Oncol.

